# Measurement of Exhaled Nitric Oxide in Cirrhotic Patients with Esophageal and Gastric Varices

**DOI:** 10.1155/2019/9673162

**Published:** 2019-11-03

**Authors:** Xiaoquan Huang, Souksavanh Thansamay, Kaiqi Yang, Tiancheng Luo, Shiyao Chen

**Affiliations:** ^1^Department of Gastroenterology and Hepatology, Zhongshan Hospital, Fudan University, 200032 Shanghai, China; ^2^Department of Gastroenterology, Mittaparb Hospital, Vientiane, Laos

## Abstract

*Background* and aims. This study aimed to detect exhaled nitric oxide (eNO) level in cirrhotic patients and explore the correlation between eNO levels and the severity of cirrhosis. *Methods*. Patients were enrolled to analyze the relationship of eNO with noncirrhosis, cirrhosis, and complications of decompensated cirrhosis. We explored the potential predictive values of eNO in different states of cirrhosis. *Results*. The eNO levels were significantly increased in cirrhotic patients compared with noncirrhotic patients (14 (10–18) vs 8 (6–13) ppb, *P* < 0.001). The eNO level was increased in those with ascites (15 (14–22) vs 13 (10–18) ppb, *P*=0.026), with portal vein thrombosis (19.5 (11.75–22) vs 13.5 (10–17) ppb, *P*=0.032), or with the mucosal red-color sign of esophageal and gastric varices (EGV) (16.5 (10–21.75) vs 13 (10–14.75) ppb, *P*=0.041). Among cirrhotic patients undergoing hepatic venous pressure gradient (HVPG) measurement, the eNO level was significantly increased in the high-HVPG group (HVPG >12 mm Hg) compared with the low-HVPG group (6 mm Hg ≤ HVPG ≤ 12 mm Hg) (15 (11.75–19.25) vs 10 (8–14) ppb, *P*=0.011). *Conclusions*. The eNO level was increased in cirrhotic patients, especially in those complicated with ascites, portal vein thrombosis, mucosal red-color sign of varices, and high HVPG.

## 1. Introduction

Cirrhosis is the end-stage chronic liver disease due to a variety of etiologies. Hypohepatia, portal hypertension (PHT), and various complications may occur in decompensated cirrhosis, including esophageal and gastric variceal bleeding, portal vein thrombosis (PVT), spontaneous bacterial peritonitis, and hepatic encephalopathy, severely affecting quality of life [1]. The increase in portal pressure in cirrhotic patients leads to dilatation of visceral arterial blood vessels of the whole body and hyperkinetic circulatory state accompanied by portosystemic collateral circulation. Given decreased liver metabolism, accumulation of toxins, increased permeability of the intestinal wall, damaged intestinal motility, and alteration and translocation of the intestinal flora, endotoxins and other gut-derived metabolites can directly stimulate blood vessels in the body or stimulate cytokines to produce nitric oxide synthase (NOS) and increase in vivo synthesis and release of nitric oxide (NO) [2]. NO is a novel signaling molecule involved in inflammation and tissue damage, which can dilate visceral blood vessels, increase visceral blood flow, and aggravate PHT. The increase in inflammatory cytokines and endotoxins in the circulation of cirrhotic patients can stimulate pulmonary vascular endothelial cells to produce small-molecule NO, which is exhaled outside the body from the respiratory tract [3]. Approximately 5%–30% of cirrhotic patients develop intrapulmonary vascular dilation and abnormal oxygenation, which is asymptomatic in the early stage or present as occult dyspnea [4].

For cirrhotic patients, the increased expression of NOS and elevated NO concentration in peripheral blood are associated with a decrease in the inactivating effect of the liver on endotoxins and increased concentration of endotoxins in the blood circulation. However, the level of serum NO is estimated mainly by detecting its metabolites nitrate and nitrite, and the results can be inaccurate [5, 6]. NO is formed in vivo from L-arginine and oxygen under the catalysis of NOS. In cirrhotic patients, the increased NO levels in pulmonary small airways and alveolar regions can lead to an increase in the eNO concentration [7–9]. The increase in eNO is more common in patients with decompensated cirrhosis, such as intrapulmonary vascular dilatation complicated with hyperdynamic circulatory syndrome and hepatopulmonary syndrome. The excessive elevation of eNO is predominantly associated with an increase in the release from pulmonary vascular endothelial cells, airway epithelial cells, and peripheral inflammatory cells [2]. The concentrations of eNO from different sites, such as central airway and alveoli, can be detected using different expiratory flows and operational formulas [8, 10, 11]. The eNO test is a noninvasive, simple, and economical method that reflects systemic inflammatory state and endothelial cell function, which may be closely related to the progression of cirrhosis [7, 12, 13]. Therefore, this study aimed to analyze the relationship of the eNO level with the severity of liver cirrhosis and evaluate the predictive value of eNO as a noninvasive marker for HVPG in patients with cirrhotic portal hypertension (CPH).

## 2. Materials and Methods

### 2.1. Participants

From June 1, 2017, to January 31, 2018, patients aged 18–75 years admitted to the Zhongshan Hospital, Fudan University, due to CPH with esophageal and gastric varices (EGV), who received endoscopic therapy, were enrolled. All patients underwent portal venous computed tomography angiography (CTA) and endoscopy with confirmed cirrhotic portal hypertension. The exclusion criteria were as follows: (1) patients complicated with lung diseases, including asthma, lung infection, chronic obstructive pulmonary disease, or chronic inflammation revealed by chest imaging, pleural effusion, etc.; (2) patients complicated with tumor; (3) patients with smoking habits within one year; and (4) patients who did not cooperate in completing the test. A total of 25 patients who were hospitalized because of intestinal polyps and gastric submucosal tumors, with no history of cirrhosis and malignant tumors, and did not met with the exclusion criteria above, were included as controls. The study protocol conformed to the guidelines of the Declaration of Helsinki and was approved by the Medical Ethics Committee of Zhongshan Hospital, Fudan University (B2015-113R). All study subjects signed an informed consent acknowledging the purpose associated with the endoscopic and eNO examination.

### 2.2. CTA and Endoscopic Examination

Cirrhosis-induced portal hypertension and the presence of PVT were confirmed by computerized tomography angiography (CTA), and the presence of gastroesophageal varices was confirmed by upper endoscopy. A routine endoscopic examination was performed after an overnight fast. All cirrhotic patients underwent propofol sedation for endoscopic examination. Endoscopy was performed using the electronic endoscope GIF-XQ260 (Olympus, Tokyo, Japan) to determine the status of varices. The form, red-color signs, and esophagogastric varices were graded according to the previous reports [14–16].

### 2.3. HVPG Measurements

Patients underwent local anesthesia, and a 7F catheter sheath was placed in the right internal jugular vein after puncturation. A 4F Cobra catheter and a VER guide wire (402–607x, the United States, Johnson & Johnson Cordis) were inserted through the catheter sheath into inferior vena cava, which was exchanged to a stiff guide wire (RF *∗* PA35263M, Japan, Terumo), and then a balloon catheter (Synergy, the United States, Boston) was inserted into hepatic vein. The balloon, 10 mm in diameter and 4 cm in length, was placed in hepatic vein, 2 cm next to the opening of inferior vena cava. A pressure transducer (42584, the United States, ICU) was connected. FHVP (free hepatic venous pressure) was measured after zero calibration. When the hepatic vein was occluded by the balloon, WHVP (wedged hepatic venous pressure) was measured after repeated zero calibration. Each measurement was recorded after the numerical fluctuations has tended steady for more than 30 seconds, and each WHVP and FHVP was measured three times to determine the average value. HVPG was the difference between WHVP and FHVP. Pressure was applied at the site of puncturation for 15–20 minutes with close observation after the procedure.

### 2.4. Measurement of eNO

The concentration of eNO in humans was measured using a Nano-Coulomb Breath Analyzer (Shangwo Biotechnology Co., Ltd., Jiangsu, China), and the unit of eNO is ppb (parts per billion) (1 ppb = 1 × 10^−9^ mol/L). The eNO level was measured based on the criteria recommended by the American Thoracic Society (ATS)/the European Respiratory Society (ERS) [10, 17]. All the included subjects were inpatients, and their meals were served by the hospital without food that influences NO readings. After fasting for 3 h, the patient was placed in the sitting position, and the appropriate height and position were maintained with exhalation. The eNO level was measured by online breath sampling or offline airbag sampling. During the online measurement, a disposable expiratory filter tip was used to tighten the lip. Air was exhaled through the mouth with efforts with an expiratory flow rate of 200 mL/s after inhaling air through the filter tip, and animation software was used to regulate the exhalation speed. After the exhalation, the device analyzed the results by itself. During the offline measurement, the patient was placed in the sitting position, and the blowing method was consistent with the online one. Using the flow rate controller, the exhalation was conducted at an average flow rate of 200 mL/s ± 10%, and the gas was retained in the airbag, which was then detected by the device immediately.

### 2.5. Statistical Methods

The categorical variables were presented as numbers (percentage) and compared with using the chi-square test. The continuous variables were reported as the mean ± standard error when the values were normally distributed, and median with others. The Student unpaired *t* test was used to evaluate normally distributed continuous variables, and the Mann–Whitney *U* was employed to other variables. A *P* value less than 0.05 was considered statistically significant. Statistical analyses and mapping were performed using SPSS 24.0 (SPSS Inc., IL, USA) and GraphPad PRISM 7.0 (GraphPad Software, CA, USA).

## 3. Results

### 3.1. General Data of Patients

From June 2017 to January 2018, a total of 164 patients with CPH underwent the detection of eNO. Further, 70 were excluded, including 28 with abnormalities on imaging examinations of the lung, 1 with idiopathic portal hypertension, 1 aged more than 75 years, 23 with smoking habit, 4 complicated with tumors, and 10 did not cooperate in completing the detection. Finally, 94 eligible cirrhotic patients were included. Of the 94 cirrhotic patients with EGV, 52 were male (55.3%) with a median age of 54.4 years; most of them had hepatitis B cirrhosis (63, 67.02%). In addition, 7 (7.45%) were complicated with hypertension and 11 (17.70%) with diabetes. Further, 76 (80.9%) had Child-Pugh grade A, 16 (17%) grade B, and 2 (2.1%) grade C. Also, 18 (19.5%) were complicated with PVT, 15 (16.0%) with a portal-systemic shunt, and 17 (18.09%) with portal hypertensive gastropathy (Table 1). The gender, age, and other baseline indicators were basically the same for the cirrhosis and the noncirrhosis groups. Patients with cirrhosis had significant higher levels of eNO compared with the controls (14 (10–18) ppb vs 8 (6–13) ppb, *P* < 0.001), as shown in Table 2.

### 3.2. Relationship of eNO with Relevant Characteristics of CPH in Cirrhotic Patients

**Figure 1:**
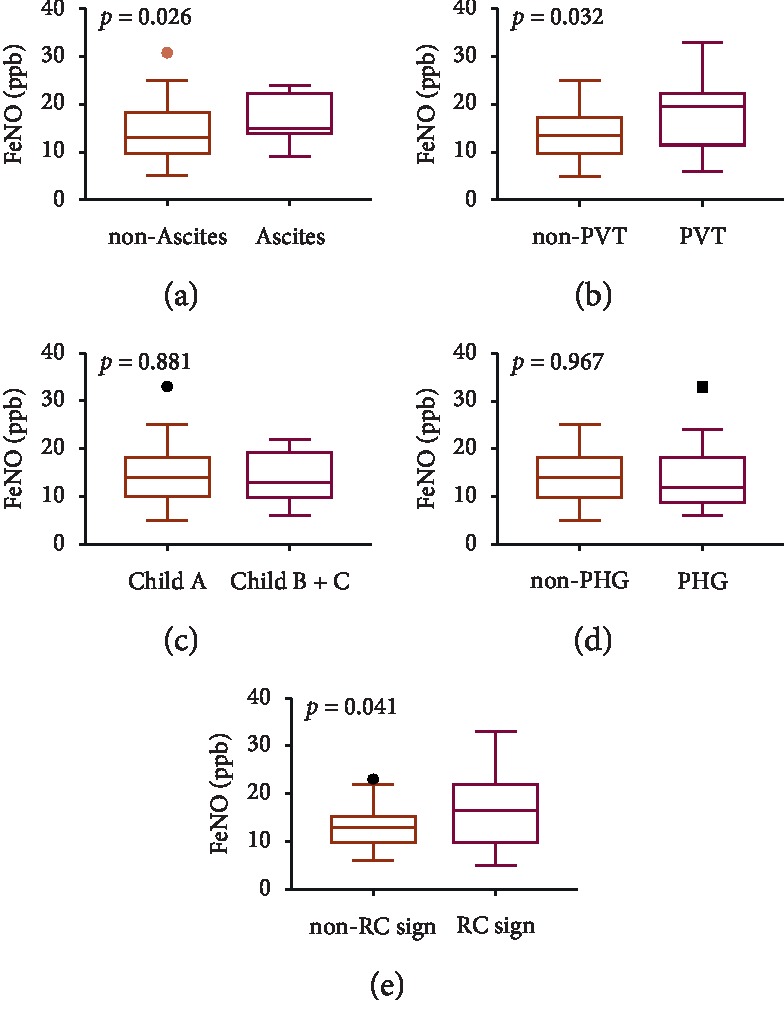
eNO values. There are statistically significant differences observed between eNO levels of the patients with ascites and without ascites (*P*=0.0253), with portal vein thrombosis (PVT) and without PVT (*P*=0.0311), and patients with red-color sign and without red-color sign (*P*=0.0413).

The eNO level was significantly increased in cirrhotic patients with ascites compared with those without ascites (15 (14–22) ppb vs 13 (10–18) ppb, *P*=0.026). The eNO level was significantly increased in cirrhotic patients with PVT compared with those without PVT (19.5 (11.75–22) ppb vs 13.5 (10–17) ppb, *P*=0.032). According to the Child-Pugh score, cirrhotic patients were classified as grade A vs grade B or C. No significant difference in the eNO level was observed between the two groups (14 (10.25–18) vs 14 (10–19), *P*=0.881). No statistically significant difference in the eNO level was found between patients with portal hypertensive gastropathy and those without gastropathy (12 (9–18) ppb vs 14 (10–18) ppb, *P*=0.967). In addition, the level of eNO in the two groups was 16.5 (10–21.75) ppb vs 13 (10–14.75) ppb (*P*=0.041), statistically significant difference between patients with or without the red-color sign of esophageal or gastric varices (Figure 1, Table 3).

### 3.3. HVPG and eNO

A total of 57 cirrhotic patients underwent hepatic venous pressure gradient (HVPG) measurement. They were assigned into two groups according to HVPG: high-HVPG group (*n* = 38) (HVPG >12 mm Hg) and low-HVPG group (*n* = 19) (6 mm Hg ≤ HVPG ≤ 12 mm Hg). A statistically significant difference in the eNO level was observed between the two groups (15 (11.75–19.25) ppb vs 10 (8–14) ppb, *P*=0.011).

## 4. Discussion

This study showed that compared with noncirrhotic patients, the eNO level in patients with CPH having EGV was significantly increased, which was consistent with the eNO level detected in the blood in previous findings [2, 8]. For those with a higher Child-Pugh score, who were complicated with ascites, PVT, and mucosal red-color sign of EGV, the level of eNO was significantly increased. As a convenient, economical, and rapid noninvasive tool, the measurement of eNO has clinical significance correlated with the severity of cirrhosis.

In cirrhotic patients complicated with ascites, the eNO level was significantly increased. NO has a strong vasodilative activity, which can decrease vascular contraction functions via factors such as endothelin and angiotensin. Previous studies suggested that a higher flow of 200 ml/s is correlated with alveolar concentration of nitric oxide (CANO) where eNO is mainly dependent on alveolar NO concentration in patients with cirrhosis. [11, 18] The eNO and plasma NO levels were increased in cirrhotic patients. [11] This resulted in visceral and peripheral vascular dilation and changes in peripheral vascular resistance. Also, the renin-angiotensin-aldosterone system was activated through the body's regulatory response, leading to retention of water and sodium. At the same time, reduced synthesis of albumin, increased portal vein pressure, and ascites were observed due to hypohepatia. Experimental studies have also suggested [19] that plasma NO levels are associated with the occurrence of ascites in cirrhotic patients and also have a relationship with the changes in visceral blood circulation.

PVT is not uncommon in patients with cirrhosis and influences the prognosis of cirrhosis [20]. Splenectomy and the use of nonselective beta-blockers (NSBBs) are associated with increased risk of PVT in cirrhotic patients [21, 22]. This study found that for patients complicated with PVT, the eNO level was significantly increased, which might be because patients with PVT were often complicated with intestinal microecological changes, activation of systemic inflammation, and low-concentration endotoxemia [23]. No study to date has investigated the relationship between PVT and eNO. Hence, further studies are needed to explore the correlations between eNO and PVT in cirrhotic patients who had splenectomy and or use of NSBBs.

All the cirrhotic patients underwent gastroscopy. The study suggested a significant increase in the eNO level in patients with the mucosal red-color sign of EGV, which was an independent predictor of the increased risk of variceal bleeding [24]. By measuring the eNO level, the mucosal red-color sign of varices may be predicted, which can indirectly reflect the risk of variceal bleeding in cirrhotic patients with gastroesophageal varices. However, this study demonstrated no significant difference in the eNO level between cirrhotic patients with portal hypertensive gastropathy and those without gastropathy. When the portal vein pressure increased, the blood flow from the gastric vein to the portal vein reduced. This led to edema, microcirculatory blood flow disorder, and mucosal barrier damage in the gastric mucosa, thus resulting in portal hypertensive gastropathy. Previous studies suggested that serum NO levels were significantly increased in patients with portal hypertensive gastropathy, which was associated with the occurrence of portal hypertensive gastropathy [25]. However, the relationship of these levels with portal hypertensive gastropathy was not reported. Therefore, further studies should be conducted to explore the relationship of eNO with portal hypertensive gastropathy.

Patients undergoing HVPG measurement were assigned to the low-HVPG and high-HVPG groups. The results showed that the eNO level was significantly increased in the high-HVPG group. Currently, HVPG is the gold standard for the clinical measurement of portal vein pressure. Generally, HVPG indicates the presence of portal hypertension when it is greater than 5 mm Hg, and HVPG suggests a significantly increased risk of EGV bleeding when it is greater than 12 mm Hg [24]. Currently, no study has explored the association between the cirrhotic portal vein pressure and eNO level. The results of this study suggested that eNO could reflect the portal vein pressure to some extent and could be used as a noninvasive detection indicator for the portal vein pressure. The increase in the eNO level was associated with the severity of CPH, which might also indirectly indicate the risk of EGV bleeding.

This study had some limitations. The measurement of eNO requires a certain degree of cooperation. However, cirrhotic patients with hepatic encephalopathy had sleepiness, coma, and so forth and hence could not cooperate in completing the measurement. Therefore, the present study did not include this patient population. Most cirrhotic patients included in this study were those with Child-Pugh grades A and B. Patients with hepatic encephalopathy were excluded, which might not accurately reflect the relationship between the Child-Pugh classification and eNO level. At the same time, multiple measurements of eNO were not conducted for the same patient; however, previous studies suggested that patients had stable eNO levels on different dates and at different times on the same date [26].

In conclusion, the eNO level was increased in cirrhotic patients with EGV and significantly increased in cirrhotic patients complicated with ascites, PVT, mucosal red-color sign of EGV, and high portal vein pressure. The eNO level is associated with the severity of CPH and may be used as a predictor of the high HVPG. Further studies with large sample size are needed to further demonstrate the predictive value of the eNO level for the portal vein pressure and HVPG in cirrhotic patients with EGV.

## Figures and Tables

**Table 1:** Clinical features and serum biochemical values in the 94 patients with liver cirrhosis.

	Patients (*n* = 94)
Etiology of cirrhosis	
Hepatitis B or C	64 (68.9%)
PBC or AIH	9 (9.57%)
Alcoholic	5 (5.3%)
Schistosomiasis	4 (4.3%)
Cryptogenic	12 (12.8%)
Splenectomy	23 (24.5%)
Portal vein thrombosis^a^	18 (19.1%)
Portal vein	15
Splenic vein	2
Superior mesenteric vein	6
Portal-systemic shunts	10 (25.6%)
Ascites (mild/severe)	8(8.5%)/11(11.7%)
Child-Pugh grade (A/B/C)	76 (80.9%)/16 (17.0%)/2(2.1%)
MELD	9.73 ± 0.27
HVPG (*n* = 63, mm Hg)	14 (8–20)
Esophageal varices	58 (61.7%)
Gastric varices	37 (39.4%)
Previous endoscopic treatment	70 (74.5%)
Average number of previous endoscopic treatments	2 (1–3)
Carvedilol	16 (17.0%)

PBC, primary biliary cirrhosis; AIH, autoimmune hepatitis; mean ± SEM; median (25%–75%). ^a^5 patients have multiple sites of portal vein thrombosis.

**Table 2:** Characteristics and exhaled nitric oxide between the cirrhosis and the noncirrhosis groups.

	Cirrhosis (*n* = 94)	Non-cirrhosis (*n* = 25)	*P* Value
Sex (male)	52 (55.3%)	9 (36%)	0.12
Age (years)	54.4 (44.4–63.0)	52.0 (40.3–57.2)	0.085
Height (cm)	166.15 ± 0.79	163.48 ± 1.22	0.108
Weight (kg)	61.35 ± 1.06	59.40 ± 2.70	0.507
BMI (kg/m^2^)	22.16 ± 0.31	22.05 ± 0.79	0.905
SpO2 (%)	98.46 ± 0.078	98.48 ± 0.15	0.906
eNO (ppb)	14 (10–18)	8 (6–13)	<0.001^*∗*^

**Table 3:** Relationship of eNO with relevant characteristics of cirrhotic portal hypertension.

	Number (%)	eNO (ppb)	*P* Value
Ascites			0.026^*∗*^
Absent	75 (79.79%)	13 (10–18)	
Present	19 (20.21%)	15 (14–22)	
Portal vein thrombosis			0.032^*∗*^
Absent	76 (80.85%)	13.5 (10–17)	
Present	18 (19.15%)	19.5 (11.75–22)	
Child-Pugh grade			0.881
A Class	76 (80.85%)	14 (10.25–18)	
B or C class	18 (19.15%)	13 (10–19)	
Portal hypertensive gastropathy			0.967
Absent	77 (81.91%)	14 (10–18)	
Present	17 (18.09%)	12 (9–18)	
Mucosal red-color sign of varices			0.041
Absent	46 (48.94%)	13 (10–14.75)	
Present	48 (51.06%)	16.5 (10–21.75)	

## Data Availability

The data used to support the findings of this study are included within the article.
